# Hydrogen-Induced Anisotropy in Single-Crystal Elastic Constants of 304L Stainless Steel via In Situ Neutron Diffraction and Kröner Modeling

**DOI:** 10.3390/ma19132796

**Published:** 2026-07-01

**Authors:** Byungrok Moon, Baek-Seok Seong, Donghyeon Choi, Jimin Nam, Jungbin Park, Seung-Gun Lee, Wanchuck Woo, Hobyung Chae, Namhyun Kang

**Affiliations:** 1 Department of Materials Science and Engineering, Pusan National University, Busan 46241, Republic of Korea; br.moon@pusan.ac.kr (B.M.);; 2 Hydrogen Materials Research Center, Korea Institute of Materials Science, Changwon 51508, Republic of Korea; leesg@kims.re.kr; 3 Neutron Science Division, Korea Atomic Energy Research Institute, Daejeon 34057, Republic of Korea; chuckwoo@kaeri.re.kr (W.W.);; 4 Institute of Materials Technology, Pusan National University, Busan 46241, Republic of Korea

**Keywords:** hydrogen embrittlement, 304L stainless steel, neutron diffraction, Kröner modeling, single-crystal elastic constants, elastic anisotropy

## Abstract

Although hydrogen embrittlement mechanisms focus predominantly on the plastic deformation regime, the fundamental effect of interstitial hydrogen on the elastic regime remains elusive. The elastic behavior due to hydrogen is critical because lattice alterations drive microstructural instabilities and macro-failure. This work aims to determine the hydrogen-affected single-crystal elastic constants and anisotropy of 304L stainless steel and link them to dislocation-mediated embrittlement mechanisms. Using in situ neutron diffraction and the Kröner model, this study derived, for the first time, the single-crystal elastic constants ($C_{ij}$) of 304L austenitic stainless steel. Hydrogen charging expanded the lattice constant by ~0.7% (from 3.558 Å to 3.583 Å) and selectively increased $C_{11}$ and $C_{12}$ while leaving $C_{44}$ nearly unchanged. Consequently, while bulk polycrystalline Young’s and shear moduli remained invariant, Zener’s anisotropy and Poisson’s ratios increased. Hydrogen reduced the shear modulus of the {111}<110> slip system by ~8.3% and the Peierls–Nabarro stress by approximately 38%. The experimental derivation of single-crystal elastic moduli proved that lattice-scale modifications selectively enhanced volumetric stiffness while lowering the slip-direction shear modulus. Coupled with hydrogen-induced lattice expansion, these findings validate the theoretical volumetric and modulus components of the hydrogen-enhanced localized plasticity mechanism, thereby elucidating its fundamental origin.

## 1. Introduction

Hydrogen embrittlement (HE) in structural alloys has been investigated across scales, from fundamental dislocation nucleation and dynamics [1,2,3] to macro-scale mechanical performance and fracture toughness in welds for H storage and offshore applications [4,5,6,7]. Complementary studies have examined H diffusion, trapping, and embrittlement susceptibility modulated by microstructure and alloying in bainitic, high-Mn, maraging, tempered martensitic, and austenitic steels [8,9,10,11,12,13]. The principal mechanistic frameworks governing the deformation regime are the H-enhanced localized plasticity (HELP) model, which accounts for enhanced dislocation mobility and slip localization [2,3], and the H-enhanced decohesion (HEDE) model, which describes reduced interfacial cohesion leading to cracking [6,7,14]; both often operate together in welded high-strength steels, with microstructure (e.g., ε-martensite or precipitates) strongly influencing crack path and resistance [6,9,11,14].

However, the fundamental impact of interstitial hydrogen on the elastic regime, specifically on the single-crystal elastic constants ($C_{ij}$), remains highly controversial and experimentally unknown. This knowledge gap is critical because lattice-level elastic alterations govern the subsequent stress fields, dislocation nucleation, and microstructural instabilities that ultimately trigger macroscale failure. Consequently, the precise experimental quantification of hydrogen-induced changes in single-crystal elasticity is a prerequisite for unveiling the early-stage driving forces of HE; however, direct measurements remain severely limited owing to the resolution constraints of conventional testing methods.

Atomistic simulations, most notably density functional theory (DFT), have been widely utilized to predict single-crystal elastic constants ($C_{11}$, $C_{12}$, and $C_{44}$) in hydrogen environments, offering valuable atomistic insights into lattice-level behaviors [15,16,17]. However, these computational approaches inherently rely on absolute zero-K constraints or athermal approximations, which limit their capacity to fully simulate temperature-dependent thermal vibrations and the dynamic behavior of hydrogen atoms [15,16,17]. Consequently, although they provide a foundational baseline for pristine lattice responses, they fundamentally lack the capability to account for complex microstructural realities and thermal variations present in engineering alloys.

The polycrystalline specimens exhibited an elastic-plastic response of any individual grain restricted by its neighbors. Furthermore, the preferential segregation of hydrogen in both the grain interiors and grain boundaries creates highly localized stress fields. Because isolated single-crystal calculations (such as DFT) assume a completely unconstrained crystal, they inherently fail to capture the complex intergranular constraints and collective grain interactions that arise within a bulk polycrystal. Therefore, deriving effective or macroscopically averaged single-crystal elastic constants that explicitly reflect these intergranular correlations is more representative of engineering materials.

Experimentally, macro-scale ultrasonic techniques and micro-scale nanoindentation are employed to evaluate elastic constants. However, ultrasonic measurements cannot be conducted simultaneously during in situ tensile loading, precluding direct tracking of elastic constant evolution under actual deformation conditions [18]. Nanoindentation is highly localized to the extreme surface, making it unrepresentative of true bulk behavior and bulk-level hydrogen effects [19].

Neutron diffraction has emerged as an indispensable tool for experimentally isolating and tracking the internal constraints of polycrystals. While the bulk specimen comprises a randomly oriented polycrystalline matrix, neutron diffraction selectively probes specific grain families whose scattering vectors are parallel to the loading direction, tracking their orientation-specific lattice strains {hkl}. Because neutrons penetrate deep into the bulk, this technique avoids surface artifacts and directly captures how these specific and diffraction-contributing grains deform when subjected to the continuous intergranular constraints of the surrounding matrix. This provides the precise and orientation-dependent bulk data required to evaluate the statistically averaged grain behaviors that govern macroscopic elasticity.

Grain homogenization mechanics offer a robust solution to bridge this scale gap between bulk polycrystals and single-crystal constituents. The Kröner self-consistent model establishes a rigorous mathematical framework by treating each constituent grain as an ellipsoidal inclusion embedded in an effective homogeneous matrix [20,21,22]. This framework allows for the backward inversion of single-crystal elastic properties directly from the measured bulk polycrystal data, effectively reflecting the statistically averaged intergranular stress fields overlooked by conventional atomistic models.

The primary objective of this study is to experimentally quantify the H-induced changes in the single-crystal elastic constants ($C_{ij}$) and anisotropy of 304L stainless steel and elucidate their direct impact on lattice-level plastic deformation. To achieve this, experimental data obtained via neutron diffraction were applied to the Kröner model to determine the single-crystal and polycrystalline elastic constants. While combining tensile testing, neutron diffraction, and the Kröner model is an established framework for pristine materials, the distinct novelty of this work lies in applying this approach to H-charged specimens for the first time to track the dynamic evolution of $C_{ij}$. From these derived constants, the stacking fault energy (SFE), slip system shear modulus, and Peierls–Nabarro (P–N) stress factors were calculated to predict the behavior within the plastic regime how H influences plastic deformation, thereby establishing a direct correlation with the HELP mechanism. Ultimately, this multi-scale approach provides a holistic framework for understanding how fundamental lattice-level elastic alterations to govern macroscopic plastic deformation and failure mechanisms, thereby uncovering the fundamental origin of HE.

## 2. Materials and Methods

### 2.1. Materials and Characterization

A 20 mm-thick 304L stainless-steel plate was produced by hot rolling. The chemical composition of the as-received plate is given in Table 1, as determined by spark optical emission spectrometry. The hot-rolled plate was solution-annealed at 1060 °C for 1 h and subsequently water-quenched. In situ slow strain rate tensile (SSRT) testing revealed that the yield strength (0.2% offset) of the specimen was 341 MPa. Notably, an insignificant difference in the yield strength was observed between the non-charged and H-charged specimens.

The specimens were mounted using a conductive resin (PolyFast, Struers, Ballerup, Denmark) and automatically polished for 1 h using a colloidal silica suspension (OP-S NonDry, Struers, Ballerup, Denmark) on an automatic polisher (MetPrep 3x^TM^, Allied High Tech Products, Cerritos, CA, USA) equipped with an automatic fluid dispenser (RD-5x^TM^, Allied High Tech Products, Cerritos, CA, USA) under an applied force of 6 N and a rotational speed of 70 rpm. Microstructural observations were performed using a field-emission scanning electron microscope (FE-SEM; SUPRA 40VP, Zeiss, Oberkochen, Germany) equipped with an electron backscatter diffraction (EBSD) detector (Hikari CCD, EDAX, Pleasanton, CA, USA). The EBSD data were acquired at an accelerating voltage of 15 kV, a working distance of 17 mm, and a step size of 1.0 μm. For postprocessing using orientation imaging microscopy (v. 7.3.1, TSL Solutions, Kanagawa, Japan), the acquired EBSD data were subjected to grain confidence index (CI) standardization (tolerance angle 5°; minimum grain size 100 pixels; multi-row 1), followed by neighbor orientation correlation (level 4; tolerance angle 5°).

### 2.2. Gaseous Hydrogen Pre-Charging

**Figure 1:**
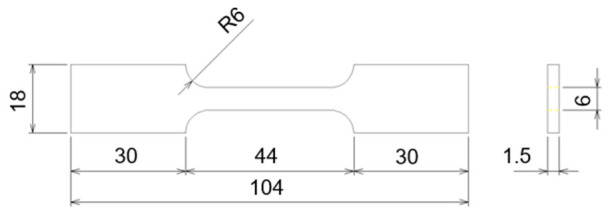
The specification of tensile testing specimens (all dimensions are in mm, R6 denotes a fillet radius of 6 mm).

Figure 1 shows the specifications of the specimens used for hydrogen pre-charging, followed by in situ neutron diffraction during stepwise tensile testing. The specimens were machined according to ASTM E8, and the final surfaces were polished using an 800-grit silicon carbide emery paper. For hydrogen pre-charging, the specimens were exposed to gaseous hydrogen at 280 °C and 16 MPa for 168 h. To minimize microstructural differences between the two conditions, non-charged specimens were subjected to the same thermal exposure (280 °C for 168 h) in the absence of H gas. Under these charging conditions, the calculated H diffusion coefficient (D) is $7.2\times 10^{-8}\;{cm}^{2}s^{-1}$ [23], yielding a characteristic diffusion distance ($x=\sqrt{Dt}$) of approximately 2 mm. Given that the specimen thickness is 1.5 mm and H diffuses simultaneously from both opposing surfaces, the maximum required diffusion distance to the center is only 0.75 mm. This ensures a fully homogeneous distribution of H throughout the entire aggregate, from the surface to the core. After hydrogen pre-charging, the hydrogen content in the specimens was quantified by thermal desorption spectroscopy (TDS). The TDS analysis was performed using a gas chromatograph (7890A, Agilent Technologies, Santa Clara, CA, USA) equipped with a heating furnace (EPKRO-22K, ISUZU Seisakusho, Sanjo, Japan). The specimens were heated at a constant rate of 200 °C h^−1^ under flowing He carrier gas, and the desorbed hydrogen was periodically monitored. The TDS peaks were deconvoluted by fitting individual hydrogen-trapping sites with an asymmetric Gaussian function (utilizing the ‘SplitGaussian’ algorithm) [24,25] using the Fityk software (v. 1.3.1) [26]. This peak deconvolution procedure was performed with reference to the TDS peak profiles and separation methodologies reported in previous studies on 304 austenitic stainless steel [27,28].

### 2.3. In Situ Neutron Diffraction Under Interrupted Tensile Loading

In situ neutron diffraction experiments were performed using a diffractometer with a residual stress instrument located at the Korea Atomic Energy Research Institute (KAERI; Daejeon, Republic of Korea), integrated with a tensile loading frame (maximum capacity of 20 kN). The scattering gauge volume was defined using incident slits of 5 mm (width) × 10 mm (height) and a detector slit of 5 mm (width). Monochromatic neutrons with a constant wavelength of 1.46 Å were obtained using a bent perfect Si (220) monochromator, and the diffracted intensities were captured by a high-resolution position-sensitive detector. The specimen was oriented 45° to the incident beam such that the loading direction (LD) was aligned within the scattering plane defined by the incident and diffracted beams. In this geometry, the scattering vectors ($\vec{Q}$) for the detector bank were parallel to the LD ($\vec{Q}∥LD$), thereby enabling the simultaneous measurement of axial strains. All measurements were conducted at a fixed specimen orientation, corresponding to a constant ψ angle (the angle between the scattering vector and the loading direction). No sample tilting was applied during the tensile test.

Tensile tests were performed at a constant strain rate of 1 × 10^−4^ s^−1^ to assess HE. Prior to each neutron diffraction measurement, tensile loading was interrupted. To acquire data exclusively within the purely elastic regime, these measurements were conducted at stress levels below 300 MPa, and individual diffraction peaks were acquired using a counting time of 135 s. After data acquisition, tensile loading was resumed at the same strain rate, during which the instantaneous gauge length was monitored using a noncontact laser extensometer.

### 2.4. Evaluation of Lattice Parameter and Lattice Strains

The peak positions and profiles obtained from neutron diffraction were determined by fitting individual diffraction peaks with a Voigt function (utilizing the VoigtA algorithm) within Fityk (v. 1.3.1) [26], followed by background subtraction in GSAS-II (v. 5.7.4) with an 8-term Chebyschev-1 polynomial [29]. Subsequently, the lattice parameters of the 304L specimens were determined using the Cohen method [30,31]. Equation (1) represents the linearized form of Bragg’s law used for this analysis, where χ and δ are defined as $h^{2}+k^{2}+l^{2}$ and $10{sin}^{2}2\theta$, respectively. By incorporating ${sin}^{2}\theta$ as the dependent variable, a three-dimensional regression model is established.

(1) $${sin}^{2}\theta =\frac{\lambda^{2}}{4a^{2}}\left(h^{2}+k^{2}+l^{2}\right)+D{sin}^{2}2\theta =S\chi +T\delta$$

For the experimental data points, the optimal fitting plane is determined by solving Equation (2) using the least-squares method. As a result, the coefficients S and T represent the partial slopes with respect to χ (corresponding to $\lambda^{2}/4a^{2}$) and δ (corresponding to D/10), respectively.

(2) ∑χsin2θ∑δsin2θ=∑χ2∑χδ∑χδ∑δ2ST

Consequently, the lattice parameter a is derived using Equation (3).

(3) $$a=\frac{\lambda }{2\sqrt{S}}$$

The lattice strain ($\varepsilon_{\left\{hkl\right\}}^{i}$) for a specific grain family was calculated according to Equation (4), which is based on the interplanar spacing ($d_{\left\{hkl\right\}}^{i}$) measured along the scattering vector under an applied load and normalized by its corresponding stress-free reference value ($d_{\left\{hkl\right\}}^{0}$) [32,33].

(4) $$\varepsilon_{\left\{hkl\right\}}^{i}=\frac{d_{\left\{hkl\right\}}^{i}-d_{\left\{hkl\right\}}^{0}}{d_{\left\{hkl\right\}}^{0}}$$

### 2.5. Derivation of Diffraction Elastic Constants (DECs) and Single-Crystal Elastic Constants (C_ij_)

To establish a consistent link between single-crystal and macroscopic elastic behaviors, mechanical constraints among the grains must be incorporated. The Voigt and Reuss approximations serve as theoretical upper and lower bounds, respectively [20,34,35,36]; the former assumes a uniform strain state (iso-strain) across all grains, while the latter assumes a uniform stress state (iso-stress). However, these models often fail to satisfy either stress equilibrium or strain continuity at grain boundaries [20,21,22]. In contrast, the Kröner self-consistent model satisfies these boundary conditions by explicitly accounting for the elastic interactions between an individual grain and its surrounding polycrystalline matrix [20,21,22]. In this study, the Kröner approach was utilized to derive grain-family-specific DECs—namely Young’s modulus ($E_{\left\{hkl\right\}}$), Poisson’s ratio ($\nu_{\left\{hkl\right\}}$), and shear modulus ($G_{\left\{hkl\right\}}$)—as a function of the single-crystal elastic constants ($C_{ij}$) [20,37]. The calculation relies on the orientation factor ($\Gamma_{\left\{hkl\right\}}$), which is determined for the respective {hkl} planes using Equation (5)

(5) $$\Gamma_{\left\{hkl\right\}}=\frac{\left(h^{2}k^{2}+k^{2}l^{2}+l^{2}h^{2}\right)}{{\left(h^{2}+k^{2}+l^{2}\right)}^{2}}$$

The relationships between the $C_{ij}$ and the auxiliary elastic parameters, specifically the shear moduli (μ, η) and bulk modulus (K) are established according to Equation (6).

(6) $$\mu =\frac{\left(C_{11}-C_{12}\right)}{2}\;\eta =C_{44}\;K=\frac{\left(C_{11}+{2C}_{12}\right)}{3}$$

Following the Kröner self-consistent approach (denoted by the superscript K), the diffraction shear modulus ($G_{\left\{hkl\right\}}^{K}$) for a specific {hkl} grain family is calculated using the third-order relation presented in Equation (7).

(7) $${\left(G_{\left\{hkl\right\}}^{K}\right)}^{3}-{\alpha \left(G_{\left\{hkl\right\}}^{K}\right)}^{2}-\beta \left(G_{\left\{hkl\right\}}^{K}\right)-\gamma =0$$

The parameters α, β, and γ within Equation (7) are determined by the single-crystal elastic parameters and the $\Gamma_{\left\{hkl\right\}}$ as expressed in Equation (8).

(8) $$\alpha =\frac{1}{5}\left(2\mu +3\eta \right)-\frac{3}{8}\left[3K+4\left\{\eta +3\left(\mu -\eta \right)\Gamma_{\left\{hkl\right\}}\right\}\right]\;\beta =\frac{3}{40}\left(6K\mu +9K\eta +20\mu \eta \right)-\frac{3}{4}K\left\{\eta +3\left(\mu -\eta \right)\Gamma_{\left\{hkl\right\}}\right\}\;\gamma =\frac{3}{4}K\mu \eta$$

Based on the previously determined $G_{\left\{hkl\right\}}^{K}$, the grain-family-specific Young’s modulus ($E_{\left\{hkl\right\}}^{K}$) and Poisson’s ratio ($\nu_{\left\{hkl\right\}}^{K}$) for the polycrystal are derived according to the self-consistent formulation presented in Equation (9).

(9) $$\frac{1}{E_{\left\{hkl\right\}}^{K}}=\frac{1}{3G_{\left\{hkl\right\}}^{K}}+\frac{1}{9K}\;\frac{1}{\nu_{\left\{hkl\right\}}^{K}}=\frac{1}{E_{\left\{hkl\right\}}^{K}}/\left(\frac{1}{6G_{\left\{hkl\right\}}^{K}}-\frac{1}{9K}\right)$$

To determine the $C_{11}$, $C_{12}$, and $C_{44}$, a weighted least-squares optimization was employed. By iteratively adjusting $C_{11}$, $C_{12}$, and $C_{44}$, the theoretical reciprocal DECs ($1/E_{\left\{hkl\right\}}^{K}$) were fitted to the experimental values ($1/E_{\left\{hkl\right\}}^{EX}$) derived from the true stress-lattice strain response. The fitting quality was evaluated using the cost function ($\chi^{2}$) in Equation (10), which incorporates the standard error ($e_{\left\{hkl\right\}}^{EX}$) associated with the linear regression of each reciprocal DEC slope ($1/E_{\left\{hkl\right\}}^{EX}$) for n grain families, to assess statistically robust identification.

(10) $$\chi^{2}=\sum_{\left\{hkl\right\}}^{n}{\left\{\left(\frac{1}{E_{\left\{hkl\right\}}^{EX}}-\frac{1}{E_{\left\{hkl\right\}}^{K}}\right)/e_{\left\{hkl\right\}}^{EX}\right\}}^{2}$$

Specifically, under the assumption of macroscopic elastic isotropy—where the orientation factor $\Gamma_{\left\{hkl\right\}}$ is set to 1/5—the solution to Equation (7) converges to the macroscopic shear modulus ($G_{M}^{K}$). Subsequently, the isotropic macroscopic Young’s modulus ($E_{M}^{K}$) and Poisson’s ratio ($\nu_{M}^{K}$) are derived using the standard elastic relationships established in Equation (11).

(11) $$E_{M}^{K}=\frac{9G_{M}^{K}K}{3K+G_{M}^{K}}\;\nu_{M}^{K}=\frac{3K-2G_{M}^{K}}{2\left(3K+G_{M}^{K}\right)}$$

## 3. Results and Discussion

### 3.1. Hydrogen Trapped, Microstructural and Lattice Parameter Behaviors Due to H-Charging

**Figure 2:**
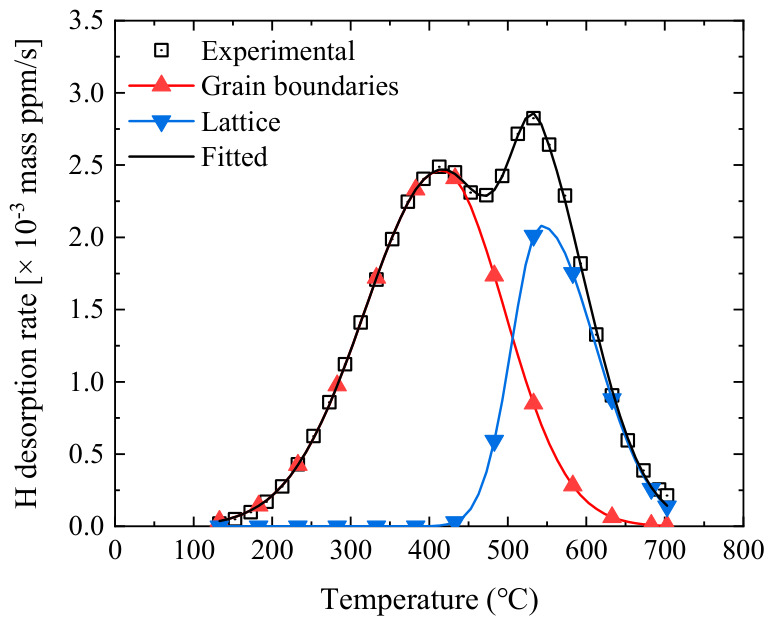
TDS spectrum with deconvoluted peaks corresponding to specific H trapping sites (grain boundaries and lattice).

Figure 2 shows a typical TDS spectrum of a hydrogen pre-charged specimen. The total hydrogen concentration of the specimen was determined to be 14.9 ± 0.1 mass ppm, as derived from two replicate measurements. Following the deconvolution of the observed peaks, the amount of hydrogen trapped at grain boundaries and within the lattice was calculated to be 9.4 ± 0.5 mass ppm and 5.5 ± 0.5 mass ppm, respectively. This TDS peak profile is similar to that observed in other TDS studies involving the H-gas charging of 304L stainless steel under comparable experimental conditions [27,28,38]. Based on the literature that performed TDS peak deconvolution [27,28], the peak in this study was deconvoluted into two distinct H trap sites: grain boundaries and lattices, as shown in Figure 2.

**Figure 3:**
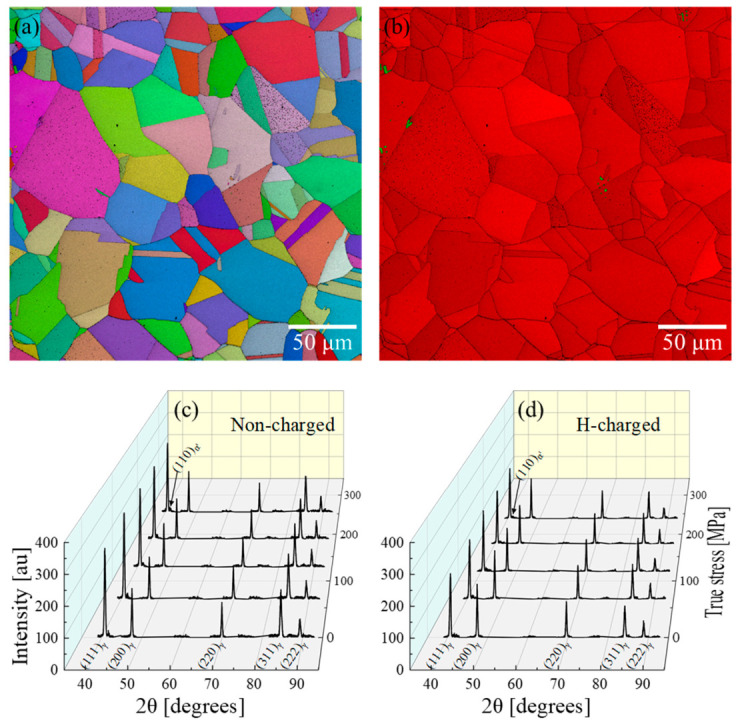
(**a**,**b**) EBSD maps of the initial microstructure for the 304L specimen, where (**a**,**b**) represent the inverse pole figure and phase maps, respectively. In (**a**), the different colors indicate both the different grains and their individual crystallographic orientations. In (**b**), red and green colors denote the austenite (γ) and α’-martensite phases, respectively. (**c**,**d**) In situ neutron diffraction patterns recorded during interrupted tensile loading for the (**c**) non-charged and (**d**) H-charged specimens.

Figure 3a,b show the typical microstructures of the 304L specimen. The average grain size was determined to be 41 ± 19 μm, calculated using the area-weighted average diameter to better reflect the volumetric contribution of larger grains to the overall mechanical properties. Furthermore, the microstructure was primarily composed of austenite (γ, 99.9 ± 0.1%) with a negligible fraction of the α’-martensite phase (0.1 ± 0.1%). The microstructure remained unchanged regardless of H-charging because the grain size and phase fraction were preserved. This is attributed to the significantly lower temperature of H-charging (280 °C) compared to the solution annealing temperature (1060 °C).

Figure 3c,d present the neutron diffraction patterns obtained under interrupted tensile loads for each specimen. Both the non-charged (Figure 3c) and H-charged (Figure 3d) specimens exhibit a predominantly austenite structure, which is consistent with the EBSD results (Figure 3b). Specifically, a trace peak of the (110)_α’_ plane was detected near the (111)_γ_ peak in both specimens. However, the intensity of the (110)_α’_ peak was extremely low compared to that of the (111)_γ_ peak during elastic deformation. Furthermore, our in situ neutron diffraction patterns (Figure 3c,d) confirmed that no macroscopic phase transformation, such as the formation of strain-induced α’-martensite, occurred after hydrogen charging or during tensile loading within the elastic regime (below 300 MPa), as the corresponding transformation peaks remained negligible and unchanged.

By using the Cohen method established in Equations (1)–(3), a least-squares regression was performed to determine the optimized coefficients and their associated standard errors. The standard error ($\varepsilon_{S}$) of the optimized coefficient (S) was propagated to the uncertainty in the lattice parameter {$\varepsilon_{a}=\left(a/2S\right)\varepsilon_{S}$}. Based on these fitting results, the lattice parameters (a) were determined as 3.558±0.008 Å and 3.583±0.006 Å for the non-charged and H-charged specimens, respectively. The introduction of interstitial hydrogen (5.5 ± 0.5 mass ppm in Figure 2) resulted in a face-centered cubic (FCC) lattice expansion of approximately 0.7% (0.025 Å). This expansion serves as the foundation for the subsequent quantification of DECs.

### 3.2. Evolution of DECs Calculated by Lattice Strain Due to H-Charging

**Figure 4:**
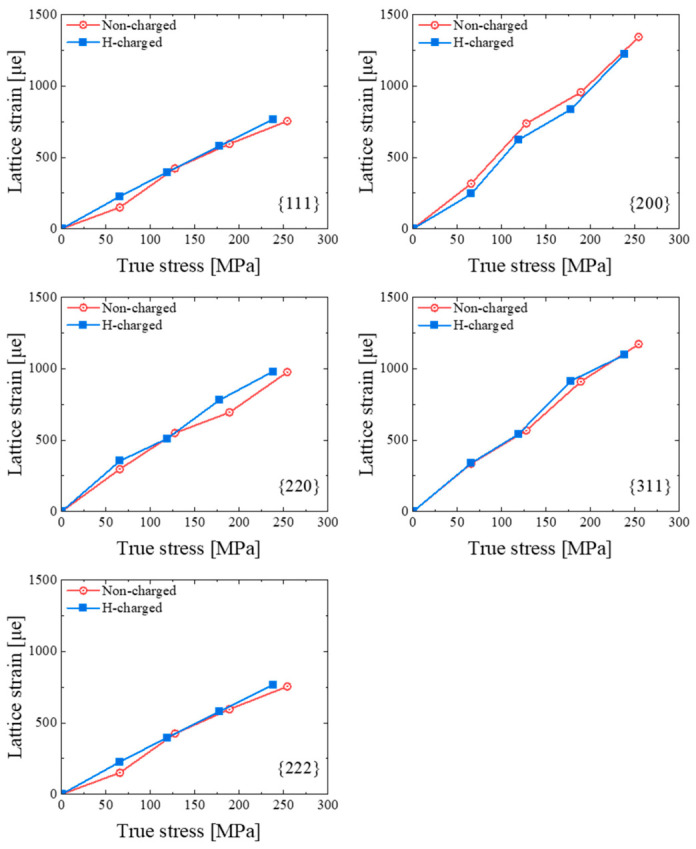
Evolution of lattice strain as a function of applied true stress for various {hkl} grain families in non-charged and H-charged 304L specimens. Red and blue symbols with corresponding solid lines represent non-charged and H-charged specimens, respectively. Peak positions for the {111} and {222} planes were corrected according to Warren’s theory. All measurements were performed at a fixed ψ (the angle between the scattering vector and the specimen loading axis).

Figure 4 illustrates the evolution of lattice strain ($\varepsilon_{\left\{hkl\right\}}$) for specific {hkl} grain families as a function of applied true stress within the elastic regime. Both specimens were subjected to a solution annealing heat treatment, with one of them being subsequently charged with hydrogen. Because both heat treatment and dissolved hydrogen significantly influence the SFE, which in turn alters the stacking fault probability and induces an apparent lattice expansion in the {111} reflection and a corresponding contraction in the {222} reflection, the true peak positions were corrected using a weighted average of the lattice parameters derived from these two reflections, following Warren’s theory [39]. This rigorous correction is essential to derive precise and reliable DECs. The calculated slopes and corresponding standard errors for all the {hkl} grain families under both testing conditions are summarized in Table 2. All linear regressions exhibited exceptional quality, with the adjusted $R^{2}$ values consistently exceeding 0.97, confirming a robust linear relationship between the applied stress and lattice strain across all diffraction planes. The magnitudes of these derived slopes increased in the order of identical {111} and {222} grain families, followed by {220}, {311}, and {200}. Figure 4 reveals negligible differences owing to H, and the actual numerical data in Table 2 further confirm that these variations are not distinct within the elastic regime.

Table 3 summarizes the DECs calculated for both specimens using Equation (10), which were derived from the lattice strain-stress relationships presented in Table 2. To assess the rigorous linear regression, only four data points were utilized, excluding the origin, to eliminate potential nonlinearities associated with initial specimen seating or instrumental artifacts [40,41]. For the {111}, {311}, and {222} planes, insignificant deviations were observed between the non-charged and H-charged specimens. However, hydrogen charging induces a discernible decrease in the DECs of the {200} and {220} planes, with reductions of approximately 3.2% and 6.7%, respectively. This demonstrates a distinct orientation dependence of the hydrogen impact, suggesting that hydrogen charging further enhances the elastic anisotropy.

### 3.3. Influence of H on Single-Crystal Elastic Constants and Polycrystal Elastic Moduli

The elastic constants serve as essential bridges between the microscopic structural features and the macroscopic mechanical responses, effectively representing the fundamental stability and stiffness of the crystal lattice. Under the framework of generalized Hooke’s law, $\sigma_{ij}=C_{ijkl}\varepsilon_{ij}$, the constitutive relationship for an FCC lattice is constrained by its cubic symmetry, which allows the stress–strain matrix to be expressed in the following form [42,43].

(12) $$\left(\begin{array}{c} \sigma_{1}\\ \sigma_{2}\\ \begin{array}{c} \sigma_{3}\\ \begin{array}{c} \tau_{1}\\ \begin{array}{c} \tau_{2}\\ \tau_{3} \end{array} \end{array} \end{array} \end{array}\right)=\left(\begin{array}{cccccc} C_{11} & C_{12} & C_{13} & 0 & 0 & 0\\ & C_{22} & C_{23} & 0 & 0 & 0\\ & & C_{33} & 0 & 0 & 0\\ & & & C_{44} & 0 & 0\\ & Sym. & & & C_{55} & 0\\ & & & & & C_{66} \end{array}\right)\left(\begin{array}{c} \varepsilon_{1}\\ \varepsilon_{2}\\ \begin{array}{c} \varepsilon_{3}\\ \begin{array}{c} \gamma_{1}\\ \begin{array}{c} \gamma_{2}\\ \gamma_{3} \end{array} \end{array} \end{array} \end{array}\right)$$

For cubic crystals, the inherent lattice symmetry dictates that only three independent elastic constants ($C_{11}$, $C_{12}$, and $C_{44}$) are required to fully characterize the elastic behavior of the system. Following the determination of the experimental DECs, the Kröner model (Equations (5)–(10)) was rigorously implemented to identify the single-crystal elastic constants. The identified $C_{ij}$ values and the resulting macroscopic elastic moduli for the non-charged and H-charged 304L specimens are summarized in Table 4. Specifically, the $C_{11}$, $C_{12}$, and $C_{44}$ values were determined to be 216, 126, and 173 GPa, respectively, for the non-charged specimen and 243, 161, and 171 GPa, respectively, for the H-charged specimen. With H-charging, $C_{11}$ and $C_{12}$ increased by approximately 13% and 28%, respectively, whereas $C_{44}$ decreased by approximately 1%. This trend is in contrast to the DECs results (Table 3), which exhibited no distinct differences induced by H. Previous studies [44,45,46] have reported pronounced differences in $C_{ij}$ even when experimental DECs remain relatively unchanged. It can be considered that the apparent variations in the properties and anisotropy of a single crystal were neutralized in the polycrystalline aggregate [47,48]. Therefore, using the Kröner model, it is worth calculating the value of $C_{ij}$ even with minor variations in the DECs, specifically for the {200} and {220} planes. The reduced $\chi^{2}$ values for the non-charged and H-charged conditions are 3.39 and 1.42, respectively, confirming a satisfactory goodness-of-fit in both instances.

Table 4 compares the $C_{ij}$ values identified in this study with those of other macroscopic elastic parameters reported in the literature. Specifically, Scott [49] utilized the impulse excitation technique (IET) for direct experimental measurements, whereas the other studies [15,16,17] relied on DFT simulations. For FCC Fe, Teus et al. [16] computed only $C_{44}$, reported a downward trend with increasing H content. Given that their DFT models assumed a significantly higher H concentration than that used in the present study, this decreasing tendency qualitatively supports our observation that $C_{44}$ remains virtually unchanged at lower H levels. Furthermore, an experimental precedent established by Scott [49] also demonstrated a subtle increase in the macroscopic elastic modulus upon H charging, capturing a qualitative trend identical to our findings, although a numerical discrepancy existed in the absolute values between the two studies. This difference is likely attributable to the distinct averaging principles of the two techniques: IET measures the bulk mechanical average across all constituent grains within the specimen, whereas neutron diffraction captures the orientation-specific averages from subsets of grains aligned parallel to the scattering vector.

Owing to the lack of comprehensive literature addressing the concurrent effects of H on $C_{11}$, $C_{22}$, and $C_{44}$ in austenitic stainless steels, the DFT data for other iron- and nickel-based systems were evaluated for comparison. In body-centered cubic (BCC) Fe, Shi et al. [15] found that a rising interstitial H concentration induces a monotonic reduction in $C_{11}$, $C_{22}$, and $C_{44}$, concurrently diminishing Zener’s ratio, bulk modulus (K), shear modulus ($G_{M}^{K}$), and Poisson’s ratio ($\nu_{M}^{K}$). Crucially, the predicted H-induced degradation of the macroscopic Young’s modulus ($E_{M}^{K}$) is in sharp contrast to the experimental enhancement demonstrated in both this study and the work of Scott [49]. Similarly, DFT simulations of FCC Ni by Hachet et al. [17] indicated that even at relatively low interstitial H concentrations, $C_{11}$, $C_{22}$, and $C_{44}$ exhibited a slight decrease, accompanied by a reduction in macroscopic moduli. Similar to the BCC Fe models, these atomistic predictions directly contradict the experimental trends captured in our and Scott’s studies [49].

Atomistic simulation studies have predominantly reported that interstitial H reduces the cohesive strength of the surrounding matrix atoms in both bcc Fe and fcc Ni structures, thereby decreasing the single-crystal elastic constants ($C_{11}$, $C_{12}$, and $C_{44}$) as well as the Young’s and shear moduli [15,16,17]. However, these simulation insights directly conflict with both the existing experimental literature [49] and the present experimental findings, which demonstrate that the Young’s modulus of austenitic stainless steels does not degrade upon H charging. Furthermore, these results are inconsistent with our discovery that H charging actually increases $C_{11}$ and $C_{12}$ while leaving $C_{44}$ virtually unchanged. This discrepancy likely arises because most atomistic simulations are conducted at 0 K or rely on athermal approximations that fail to fully capture thermal vibrations and the dynamic rearrangement of H atoms [15,16,17], ultimately leading to systematic deviations from experimental evaluations performed under ambient conditions.

Most importantly, there is a fundamental distinction between atomistic simulations and the Kröner model. Most atomistic simulations of HE are based on DFT and focus on isolated and unconstrained single crystals, thereby failing to account for the intergranular elastic interactions that inherently occur within actual polycrystals. Conversely, the Kröner model inversely calculates single-crystal elastic constants from polycrystalline neutron diffraction data while incorporating self-consistent intergranular elastic interactions, thereby offering a far more realistic representation of the polycrystalline environment. As illustrated in Figure 2, H resides extensively not only within the grain interior but also along the grain boundaries. Consequently, using the Kröner model to evaluate the average single-crystal elastic constants under the influence of intergranular elastic interactions is significantly more appropriate and physically rigorous than using DFT calculations.

Consequently, rather than diminishing the cohesive strength of the surrounding Fe atoms within the lattice, interstitial H in 304L austenitic stainless steel primarily drives local lattice distortion and selective volumetric stiffening. The introduced interstitial H induces a lattice expansion (0.7%), directly altering the equilibrium interatomic distance and the underlying atomic bonding strength. This behavior is strongly evidenced by the increases in $C_{11}$ and $C_{12}$, invariance of $C_{44}$, and the increase in the bulk modulus (K), which governs the resistance to volumetric changes. Therefore, the H-induced local lattice distortion is not a confounding artifact to be excluded, but rather the primary physical driving force that intrinsically dictates the observed alterations in single-crystal elastic constants. Hence, it is reasonable to deduce that H selectively enhances volumetric lattice stiffness.

#### 3.3.1. Response of Single-Crystal Elastic Constants

The elastic constant $C_{11}$ represents the longitudinal stiffness of the {100}<100> system, where the normal stress and strain are aligned along the principal axes. Meanwhile, the $C_{12}$ characterizes the transverse elastic coupling between the orthogonal <100> and <010> directions, representing how stress applied along one axis influences the deformation in perpendicular directions [50]. In this study, hydrogen charging resulted in a significant increase in the $C_{11}$ and $C_{12}$ values. In contrast, $C_{44}$, which denotes the shear modulus for the {100}<010> system [50], exhibited only a marginal change, indicating that the impact of hydrogen on the pure shear resistance was relatively minor.

Zener’s ratio (A), defined by the following equation, is a fundamental metric used to quantify the degree of elastic anisotropy in cubic crystals [20]:

(13) $$A=\frac{2C_{44}}{C_{11}-C_{12}}$$

The primary purpose of evaluating Zener’s ratio in this study is to characterize how interstitial hydrogen modifies the directional dependence of the elastic response of the lattice. In this study, H-charging increased Zener’s ratio from 3.8 to 4.2, signifying a marked elevation in elastic anisotropy (Table 4). Heightened anisotropy is expected to trigger three distinct and synergistic mechanisms that collectively increase the hydrogen embrittlement susceptibility.

(1) Magnification of elastic mismatch: Intensified anisotropy increases the elastic mismatch between adjacent grains, leading to a localized stress concentration at the grain boundaries that can significantly facilitate intergranular crack initiation.
(2) Promotion of strain localization: Pronounced discrepancies in stiffness across various crystallographic planes promote the localization of deformation, causing the strain to concentrate preferentially on the primary {111} slip planes in the subsequent plastic deformation.
(3) Reduction in SFE: An elevated Zener’s ratio (A) has been documented to correlate with a reduction in SFE using the following equation [51,52], a phenomenon that subsequently facilitates the formation of strain-induced martensite and further degrades the ductility of the material:

(14) $$\gamma =\frac{K_{111}\omega_{0}G_{\left(111\right)}a_{0}A^{-0.37}}{\pi \sqrt{3}}\frac{{\langle \epsilon_{50}^{2}\rangle }_{111}}{\alpha }$$

$K_{111}\omega_{0}$ equivalent to 6.6 is the proportionality constant, $G_{\left(111\right)}$ defined by $\left(C_{44}+C_{11}-C_{12}\right)/3$, is shear modulus in the (111) fault plane, $a_{0}$ is the lattice constant, the root mean square microstrain measured, ${\langle \epsilon_{50}^{2}\rangle }_{111}$, is averaged over a column 50 Å long in the [111] direction, and α is the stacking fault probability.

The simultaneous increase in both $C_{11}$ and $C_{12}$ was further reflected in the bulk modulus, $K=\left(C_{11}+2C_{12}\right)/3$, rising from 156 GPa in the non-charged state to 188 GPa upon H-charging, a 20.5% increase. Physically, this reflected the increased resistance of the crystal lattice to volumetric changes under hydrostatic pressure.

Ultimately, these findings highlight that even within the elastic regime, where H-induced effects may not be macroscopically apparent in the stress–strain curves, the fundamental single-crystal elastic constants are significantly altered. This early alteration served as a critical precursor governing subsequent plastic localization and mechanical degradation.

#### 3.3.2. Evaluation of Polycrystal Elastic Moduli

The Young’s modulus ($E_{M}^{K}$) exhibited near-invariance (from 251 to 252 GPa) owing to H-charging, governed by the competing effects of the increased K and nearly constant $G_{M}^{K}$ (Table 4). The polycrystalline shear modulus ($G_{M}^{K}$) exhibited only a marginal reduction from 102 to 99 GPa, which is consistent with the negligible change observed in $C_{44}$ at the single-crystal level. These results indicate that macroscopic elastic and shear resistance remain largely unaffected by hydrogen in the elastic regime, explaining why the standard stress–strain curves obtained from in situ SSRT show no apparent softening prior to fracture.

Upon hydrogen charging, the Poisson’s ratio ($\nu_{M}^{K}$) increases from 0.23 to 0.28 (Table 4). This increase is a direct consequence of the preferential stiffening of the bulk modulus K relative to the nearly invariant shear modulus G, as described by the relation $\nu =\left(3K-2G\right)/\left(6K+2G\right)$. The selective enhancement of the volumetric response was driven by the pronounced increases in $C_{11}$ and $C_{12}$, whereas the shear stiffness represented by $C_{44}$ remained essentially unchanged. Consequently, the elastic response of the material shifted, such that the resistance to volume change became relatively stronger than that to shape change. In a polycrystalline aggregate, such a modification of the bulk-to-shear modulus ratio (K/G) can alter the internal stress and strain partitioning among the grains during the early stages of deformation, even before extensive plastic flow develops [53]. These continuum-level changes in the elastic behavior may contribute to alterations in the plastic deformation response of the H-charged specimens.

These polycrystal moduli reveal that hydrogen selectively modifies the volumetric elastic response while preserving the shear response, a mechanistic asymmetry captured only at the single-crystal level and undetectable by conventional macroscopic mechanical testing such as SSRT.

### 3.4. Prediction of Dislocation Mobility via Single-Crystal Elastic Constants

Based on previously determined $C_{ij}$ values, the shear modulus ($G_{\left\{hkl\right\}}$) can be defined using Equation (15) [54,55].

(15) $$\frac{1}{G_{\left\{hkl\right\}}}=\frac{1-2\Gamma_{\left\{hkl\right\}}}{C_{44}}+\frac{4\Gamma_{\left\{hkl\right\}}}{C_{11}-C_{12}}=S_{44}+4\left(S_{11}-S_{12}-\frac{S_{44}}{2}\right)\Gamma_{\left\{hkl\right\}}\;S_{44}=\frac{1}{C_{44}}\;S_{11}-S_{12}=\frac{1}{C_{11}-C_{12}}$$

Using Equation (15), the shear modulus ($G_{\left\{hkl\right\}}$) was calculated for the {111}<110> primary slip system under both non-charged and H-charged conditions. The results yielded $G_{\left\{111\right\}}^{non-charged}$ and $G_{\left\{111\right\}}^{H-charged}$ values of 60 and 55 GPa, respectively, indicating that hydrogen induced a reduction of approximately 8.3% in the shear modulus of the {111} plane. These results were subsequently used to calculate the P–N stress, which represents the minimum shear stress required to displace a dislocation within the crystal lattice, as expressed in Equation (16) [56,57].

(16) $$\tau_{P-N}=\frac{2G_{\left\{111\right\}}}{\left(1-\nu \right)}exp\left(\frac{-4\pi \zeta }{b}\right)$$

where ζ is the half-width of the dislocation $\left(=d/2\left(1-\nu \right)\right)$; d is the interplanar spacing $\left(=a/\sqrt{h^{2}+k^{2}+l^{2}}\right)$; and b is the Burger’s vector for cubic crystals. Notably, this classical equation accounts exclusively for perfect dislocations and does not incorporate partial dislocations or the SFE.

For the primary slip system of the FCC lattice, the calculated $\tau_{P-N}^{non-charged}$ and $\tau_{P-N}^{H-charged}$ values are 0.198 and 0.123 GPa, respectively. The presence of interstitial hydrogen within the lattice reduces the P–N stress by approximately 38%. Although these values may be overestimated owing to the assumption of perfect dislocations [56], instead of stacking-fault-mediated partial dislocations that typically require advanced methods (e.g., semi-discrete variational P–N [58] or DFT-coupled models [59]), this classical formulation serves as a meaningful linear approximation. This calculation via Equation (16) was intended to clearly illustrate the general trend of H-induced P–N stress reduction. Since both perfect dislocations [{111}<110>] and partial dislocations [{111}<112>] glide on the exact same {111} plane, the confirmed decrease in the $G_{\left\{hkl\right\}}$ shear modulus makes it highly reasonable to predict that the P–N stress for partial dislocations will similarly decrease under the influence of H. The shear modulus and P–N stress changes in the elastic regime have direct consequences on the plastic behavior. The reduced shear modulus ($G_{\left\{111\right\}}$) led to a decrease in the SFE (Section 3.3.1) and facilitated slip initiation. A lower SFE suppressed cross-slip while promoting the predominance of planar slip. Consequently, plasticity becomes localized owing to the preferential planar slip, and the combined effects of the facilitated slip further intensify localized strain accumulation. Furthermore, the reduced P–N stress facilitated dislocation glide along the planar slip planes, thereby promoting greater dislocation accumulation in regions of localized strain. Notably, these phenomena are consistent with the HELP mechanism. Accordingly, minimizing H-induced changes in single-crystal elastic constants through alloy design may offer a viable strategy to suppress the HELP mechanism and improve the HE resistance of austenitic steels.

It is worth noting that while the Kröner model is mathematically restricted to the elastic regime, the identified H-induced alterations in $C_{ij}$ and $\tau_{P-N}$ are expected to persist into the early stages of plastic deformation. This is because dislocation-driven H transport to grain boundaries is highly limited due to low initial dislocation activity at the onset of yielding [60]. Thus, the lattice H concentration remains stable, allowing the elastic anisotropy to continuously govern the early plastic behavior.

To interpret these findings within the broader scope of HE theories, alternative mechanisms were critically evaluated against the observed $C_{ij}$ trends:

(1) Intragranular HEDE: The H-induced increase in $C_{11}$ directly disproves lattice-level decohesion within the grain interiors during the elastic regime, which would otherwise decrease tensile stiffness.
(2) Intergranular HEDE and Adsorption-Induced Dislocation Emission (AIDE): The observed $C_{ij}$ variations do not support these localized surface- or boundary-driven mechanisms, as these phenomena are mechanically independent of the bulk lattice changes measured by neutron diffraction. Specifically, AIDE is strictly governed by surface-adsorbed H that facilitates dislocation emission at the crack tip, while intergranular HEDE is reasonably explained by the concentration of H trapped at the grain boundaries, both of which operate independently of bulk lattice elasticity.
(3) HELP-mediated HEDE and Hydrogen-Enhanced Strain-Induced Vacancy (HESIV): The clear decrease in $G_{\left\{111\right\}}$ and $\tau_{P-N}$ strongly supports other theories that rely on faster dislocation movement. This includes the HESIV mechanism, where H stabilizes deformation-induced vacancies that cluster into nano-voids–a process driven entirely by active dislocations. Since our bulk elastic data prove that dislocations move more easily, they provide the core physical foundation for these dislocation-based theories. Consequently, the observed changes in bulk elastic anisotropy directly confirm the activation of the HELP mechanism.

### 3.5. Identifying the Origin of the HELP Mechanism Through Lattice-Scale Elastic Measurements

Birnbaum and Sofronis [61] theoretically proposed two elastic interactions underlying the HELP mechanism: the volumetric effect (induced volumetric strain) and the modulus effect (changes in the constitutive moduli) due to interstitial H. H-induced lattice expansion in FCC Fe-based alloys has been reported in other studies [62,63] and corroborated by the present study, providing experimental support for the volumetric effect. However, H-induced changes in elastic constants at the single-crystal level have not yet been experimentally measured. The present study addresses this gap; the 8.3% reduction in $G_{\left\{111\right\}}$ obtained in Section 3.4 provides the first experimental quantification of this modulus effect upon the H charging of 304L austenitic stainless steel. Although conventional studies have predominantly focused on the consequential phenomena of the HELP mechanism, such as the enhanced dislocation velocity observed via in situ transmission electron microscopy [3,64] and early plastic initiation detected via nanoindentation [1,2], the present study successfully elucidated the fundamental origin by directly quantifying these lattice-scale elastic modifications. Consequently, the validity of both the volumetric and modulus effects theoretically proposed by Birnbaum and Sofronis [61] has been experimentally substantiated, and the direct experimental derivation of single-crystal elastic moduli clearly validates both the H-induced lattice expansion and accompanying alterations in constitutive moduli.

## 4. Conclusions

Neutron diffraction was employed to experimentally determine the effect of hydrogen on the single-crystal elastic constants ($C_{11}$, $C_{12}$, and $C_{44}$) of 304L austenitic stainless steel using an inverse Kröner self-consistent model. The quantitative behavior of the elastic properties and the attributable impact on the plastic properties upon hydrogen charging are summarized as follows.

1. Hydrogen charging expanded the lattice constant by ~0.7% (0.025 Å), from 3.558±0.008 Å to 3.583±0.006 Å.
2. Hydrogen preferentially altered the axial elastic properties over pure shear resistance, that is, elevating $C_{11}$ and $C_{12}$ while leaving $C_{44}$ virtually unaffected, thereby enhancing the volumetric stiffness and elastic anisotropy.
3. The elevated Zener’s ratio (magnifying mismatch, planar localization, and stacking fault energy reduction) and increased bulk modulus, K, which heightened the hydrostatic volumetric resistance, collectively increased the hydrogen embrittlement susceptibility.
4. While the Young’s and shear moduli of the polycrystal remained invariant, hydrogen reduced the {111} shear modulus by ~8.3% and the Peierls–Nabarro stress by ~38%, thereby promoting planar slip and validating the lattice-level origin of hydrogen-enhanced localized plasticity (HELP).
5. Single-crystal elastic modulus measurements provide experimental evidence of both volumetric and modulus effects, fundamentally identifying the origin of the HELP mechanism.

## Figures and Tables

**Table 1:** Chemical composition of the 304L austenitic stainless steel (wt.%).

C	Cr	Ni	Mn	Mo	Si	Fe
0.02	18.1	8.0	1.6	0.1	0.4	Bal.

**Table 2:** Linear fitting results of the data presented in Figure 4, excluding the origin for each diffraction plane to ensure a purely elastic evaluation.

$1/E_{\left\{hkl\right\}}^{EX}$ $\left[\times 10^{-6}\right]$	Non-Charged Specimen (Slope ± Standard Error)	H-Charged Specimen (Slope ± Standard Error)
{111}	$3.16\pm 3.22\times 10^{-1}$	$3.13\pm 1.64\times 10^{-2}$
{200}	$5.27\pm 4.25\times 10^{-1}$	$5.42\pm 5.25\times 10^{-1}$
{220}	$3.51\pm 1.87\times 10^{-1}$	$3.76\pm 2.06\times 10^{-1}$
{311}	$4.64\pm 2.42\times 10^{-1}$	$4.65\pm 4.39\times 10^{-1}$
{222}	$3.16\pm 3.24\times 10^{-1}$	$3.13\pm 1.79\times 10^{-2}$

**Table 3:** Experimentally determined diffraction elastic constants (DECs) for non-charged and H-charged 304L specimens.

		{111}	{200}	{220}	{311}	{222}
$E_{\left\{hkl\right\}}^{EX}$ $\left[GPa\right]$	Non-charged	317	190	285	216	317
	H-charged	320	184	266	215	319

**Table 4:** Identified single-crystal elastic constants and macroscopic elastic moduli for the non-charged and H-charged 304L specimens. The $C_{ij}$ in the previous study was calculated by DFT and the remaining values were calculated from Equations (5)–(9) and (11). Scott’s study measured the moduli experimentally using IET [49].

	Single Crystal					Polycrystal			
	$C_{11}$ [GPa]	$C_{12}$ [GPa]	$C_{44}$ [GPa]	Zener’s Ratio (*A*)	K [GPa]	$E_{M}^{K}$ [GPa]	$G_{M}^{K}$ [GPa]	$\nu_{M}^{K}$ [GPa]	
In this study (Neutron diffraction)									
Non-charged (304L)	216	126	173	3.8	156	251	102	0.23	
H-charged (0.1 at. % H) (304L)	243	161	171	4.2	188	252	99	0.28	
Kevin M. Scott (Impulse excitation tech.)									[49]
Non-charged (316L and XM-19)	-	-	-	-	-	196	-	-	
H-charged (1.2 at. % H) (316L and XM-19)	-	-	-	-	-	197	-	-	
S. M. Teus et al. (DFT)									[16]
Non-charged (FCC Fe)	-	-	279	-	-	-	-	-	
H-charged (20 at. % H) (Fe_4_H/Octa.)	-	-	229	-	-	-	-	-	
H-charged (50 at. % H) (FeH/Octa.)	-	-	186	-	-	-	-	-	
Ying Shi et al. (DFT)									[15]
Non-charged (BCC Fe)	284	156	121	1.9	198	243	94	0.30	
H-charged (1.8 at. % H)	272	143	115	1.8	186	236	91	0.29	
H-charged (6.9 at. % H)	258	125	102	1.5	169	221	86	0.28	
G. Hachet et al. (DFT)									[17]
Non-charged (FCC Ni)	282	157	134	2.1	199	255	99	0.29	
H-charged (1.5 at. % H) (H0.016)	281	157	132	2.1	198	252	98	0.29	
H-charged (H_0.016_-Vac_0.016_)	269	151	130	2.2	190	245	95	0.29	

**Note:** $C_{11}$, $C_{12}$, $C_{44}$ are single-crystal elastic constants; K is bulk modulus; $E_{M}^{K}$, $G_{M}^{K}$, and $\nu_{M}^{K}$ are macroscopic Young’s modulus, shear modulus, and Poisson’s ratio, respectively, calculated using the Kröner method. DFT: density functional theory; IET: impulse excitation technique; FCC: face-centered cubic; BCC: body-centered cubic; at. % H: atomic percentage of hydrogen; Vac: vacancy.

## Data Availability

The original contributions presented in this study are included in the article. Further inquiries can be directed to the corresponding author.
